# Rotavirus P[4]G2 in a Vaccinated Population, Brazil

**DOI:** 10.3201/eid1405.071440

**Published:** 2008-05

**Authors:** Manish M. Patel, Lucia Helena de Oliveira, Ana Maria Bispo, Jon Gentsch, Umesh D. Parashar

**Affiliations:** * Centers for Disease Control and Prevention, Atlanta, Georgia, USA; † Pan American Health Organization, Washington, DC, USA

**Keywords:** Rotavirus, strains, vaccination, letter

## To the Editor

Gurgel et al. provide an early examination of postmarketing surveillance data from Brazil, one of the first countries to implement routine childhood immunization with Rotarix vaccine. In a community with reported vaccination coverage of 50%, the P[4]G2 strain was detected in all 21 rotavirus-positive stool samples identified during November 2006–February 2007. Although monitoring effectiveness of Rotarix against P[4]G2 strains is of interest, the small sample size, short duration of surveillance, and lack of a comparison group preclude firm assessment of an association between P[4]G2 predominance and vaccination.

Because Rotarix was introduced in Brazil in March 2006, most children >12 months old (66 [51%] of 129) in the study were ineligible for vaccination. Genotype P[4]G2 was the only strain identified even in older children, which suggests either a change in disease ecology from vaccination or the random circulation of P[4]G2 strains in the community. Ongoing hospital-based surveillance during 2006 in 3 regional countries that had not introduced rotavirus vaccine (El Salvador, Guatemala, and Honduras) showed that P[4]G2 was the predominant circulating strain (prevalence 68%–81%). Thus, as previously documented, the predominance of P[4]G2 strains after Rotarix introduction in Brazil could represent a natural shift unrelated to vaccination.

Evaluation of vaccine effectiveness against specific strains will allow full assessment of the public health impact of vaccination. Although the data are sparse in the study from Gurgel et al., a comparison of the odds of vaccination among rotavirus-positive (cases) versus rotavirus-negative (controls) children shows 80% vaccine effectiveness against P[4]G2 strains among infants <1 year of age, in accordance with recently published data from a controlled trial. To further elucidate vaccine impact, we are providing support for vaccine effectiveness studies in Nicaragua and El Salvador and conducting strain monitoring before and after licensure throughout Latin America.
